# Multiplexed Anodic Stripping Voltammetry Detection of Heavy Metals in Water Using Nanocomposites Modified Screen-Printed Electrodes Integrated With a 3D-Printed Flow Cell

**DOI:** 10.3389/fchem.2022.815805

**Published:** 2022-02-17

**Authors:** Guo Zhao, Thien-Toan Tran, Sidharth Modha, Mohammed Sedki, Nosang V. Myung, David Jassby, Ashok Mulchandani

**Affiliations:** ^1^ Department of Chemical and Environmental Engineering, University of California, Riverside, Riverside, CA, United States; ^2^ College of Artificial Intelligence, Nanjing Agricultural University, Nanjing, China; ^3^ Department of Bioengineering, University of California, Riverside, Riverside, CA, United States; ^4^ Materials Science and Engineering Program, University of California, Riverside, Riverside, CA, United States; ^5^ Department of Civil and Environmental Engineering, University of California, Los Angeles, Los Angeles, CA, United States; ^6^ Center for Environmental Research and Technology (CE-CERT), University of California, Riverside, Riverside, CA, United States

**Keywords:** 3D printing, screen printed electrode, heavy metals detection, anodic stripping voltammetry, flow injection

## Abstract

In this study, we present multiplexed anodic stripping voltammetry (ASV) detection of heavy metal ions (HMIs)—As(III), Cd(II), and Pb(II)—using a homemade electrochemical cell consisting of dual working, reference and counter screen-printed electrodes (SPE) on polyimide substrate integrated with a 3D-printed flow cell. Working and counter electrodes were fabricated by the screen-printing of graphite paste while the Ag/AgCl paste was screen-printed as a reference electrode (Ag/AgCl quasi-reference electrode). The working electrodes were modified with (BiO)_2_CO_3_-reduced graphene oxide (rGO)-Nafion [(BiO)_2_CO_3_-rGO-Nafion] and Fe_3_O_4_ magnetic nanoparticles (Fe_3_O_4_MNPs) decorated Au nanoparticles (AuNPs)-ionic liquid (IL) (Fe_3_O_4_-Au-IL) nanocomposites separately to enhance HMIs sensing. Electrochemical detection was achieved using square wave ASV technique. The desired structure of the flow electrochemical cell was optimized by the computational fluid dynamic (CFD). Different experimental parameters for stripping analysis of HMIs were optimized including deposition time, deposition potential and flow rate. The linear range of calibration curves with the sensing nanocomposites modified SPE for the three metal ions was from 0–50 μg/L. The limits of detection (S/N = 3) were estimated to be 2.4 μg/L for As(III), 1.2 μg/L for Pb(II) and 0.8 μg/L for Cd(II). Furthermore, the homemade flow anodic stripping sensor platform was used to detect HMIs in simulated river water with a 95–101% recovery, indicating high selectivity and accuracy and great potential for applicability even in complex matrices.

## Introduction

With the development of global industrialization, especially, in the electronic and electrical industry, increasing amounts of heavy metal ions (HMIs) are inadvertently discharged into the environment. HMIs pose a great threat to ecological and human health because of their potential for bioaccumulation and toxicity (Verma and Singh, 2005; Wuana and Okieimen, 2011; Wu et al., 2018). Among different heavy metals, Cd(Ⅱ), Pb(Ⅱ), and As(Ⅲ) are widely distributed in various environments (e.g., soil and water) with particularly high toxicity, which highlights the urgency for effective and rapid detection methods (Panov et al., 2008). Traditionally used analytical methods for the detection of HMIs include, atomic absorption spectroscopy (AAS) (Kenawy et al., 2000), atomic fluorescence spectrometry (AFS) (Wan et al., 2006), X-ray fluorescence spectrometry (XRF) (Galani-Nikolakaki et al., 2002) and inductively coupled plasma mass spectroscopy (ICP-MS) (Davis et al., 2007; Silva et al., 2009). These techniques have advantages of high sensitivity and selectivity; however, they have to be performed in a laboratory, which requires transportation of the samples to the laboratory, and use of expensive benchtop instrumentation by trained personnel. These increase the analysis costs and delay the results thereby limiting their widespread application, especially for portable and on-site detection. Additionally, these conventional instruments and techniques require time consuming intricate operating procedures and sample preconcentration steps (Bi et al., 2010).

Anodic stripping voltammetry (ASV)—an electrochemical analysis technique—is a promising method that can be used for HMIs detection with high sensitivity, selectivity and accuracy, while amenable for on-site detection and quantification when coupled to a portable potentiostat (Pandey et al., 2016). Furthermore, integrating the traditional batch mode ASV with a flow cell/system presents an attractive analytical platform for the continuous monitoring of HMIs (Becker and Locascio, 2002; Reyes et al., 2002; West et al., 2008). C. Henríquez et al. proposed an automatic multisyringe flow injection system coupled to a flow-through screen printed electrode (SPE) for the detection of Cd(Ⅱ) (Henríquez et al., 2012). Z. M. Redha et al. developed a microfluidic sensor fabricated by screen printing and injection molding for electrochemical detection of Cu(Ⅱ) and Pb(Ⅱ) (Redha et al., 2009). Sun et al. described a flow electrochemical system for the detection of Pb(Ⅱ) with a detection limit of 0.2 μg/L, showing a better sensitivity and reproducibility compared to the traditional batch mode ASV (Sun et al., 2017). However, the flow systems integrated with multiple SPEs, which can simultaneously detect Cu(Ⅱ), Pb(Ⅱ), and As(Ⅲ), need to be further developed. The combination of ASV and flow system would enable automated, on-site and near-/real-time monitoring of HMIs in water samples at low cost, which can further mitigate the spread of environmental pollution (Alpízar et al., 1997; Economou, 2010). A three-electrode system, consisting of a reference electrode (RE), a counter electrode (CE) and a working electrode (WE), is generally used for the ASV analysis, in which the WE is the most important component due to its significant influence on the detection performance as a site for electrochemical reactions. The conventional electrodes such as glassy carbon electrode and carbon paste electrode are not suitable for small-volume analysis due to their relatively large size. Thus, the development of a planar sensor device compatible with a flow cell enables ease-of-integration of the sensor into the flow injection system.

The technique of screen-printing has been considered an effective method for the fabrication of whole electrode system in a small footprint/form factor, including the counter electrodes, reference electrodes and working electrodes, with a variety of printing patterns, either consisting of individual electrodes or electrode arrays. In addition, the screen-printed electrodes (SPEs) can be fabricated at low costs with high precision, and can be easily replaced during operation. The SPEs can be fabricated on different kinds of substrate, such as glass, ceramic and flexible polymer polyimide, based on the characteristics of printing. The SPEs printed on polyimide substrates are capable of integration in a flow cell with a small volume attributed to the thin and mechanically flexible property of the substrate material. However, there are still some problems that need to be resolved when SPEs are applied in flow system (Erlenkötter et al., 2000; Leca and Blum, 2000). First, SPEs should be incorporated in a flow cell with a good adaptability and mechanical stability. Second, the sensing area on SPE should be placed strategically in the flow channel to ensure efficient electrodeposition of HMIs in the flow channel on the working electrode surface. Third, the dead volume formed in the flow cell must be eliminated by the optimization of the flow cell geometry. Fourth, reliable sealing method is required to prevent leakage between the SPE and flow cell assembly. Furthermore, the sensitivity, selectivity, and specificity of the bare SPE-based sensors need to be improved. Thus, the modification of sensing materials has been widely investigated to enhance the performance of SPE-based stripping voltammetry detection of HMIs (Chen et al., 2013; Puy-Llovera et al., 2017; Tu et al., 2018). Lately, three-dimensional (3D) printing has received a great deal of attention as one kind of rapid prototyping technology, which has been widely applied in various microscale production fields (Andrew Clayton et al., 2006; Gross et al., 2014; Kolesky et al., 2014; Murphy and Atala, 2014; Johnson et al., 2015; Ambrosi et al., 2016; Au et al., 2016). With the decreased cost of printing systems, the flow cell can be fabricated with desired shape with extraordinary freedom (Whitesides, 2006; Ge et al., 2012).

Nano-materials/composites modified electrodes for detection of heavy metals Pb(II), Cd(II), and As(III) in water by ASV in batch electrochemical cells has received a great deal of attention (Bi et al., 2010; Pandey et al., 2016; Zhao et al., 2020; Sedki et al., 2021). While highly sensitive and selective, batch cells have limitations. In this paper, we achieved multiplexed ASV detection of As(III), Cd(II), and Pb(II) using planar SPEs on polyimide substrate integrated in a 3D-printed flow cell. The employment of the disposable SPEs avoided the traditional pretreatment process frequently-used to prepare glassy carbon or carbon paste WE, but obtained a comparable sensitivity based on the modification of the nanocomposites. Compared to the batch electrochemical cells in previously published papers, including ours (Zhao et al., 2020; Sedki et al., 2021), the flow electrochemical stripping analysis reported in this study has the advantages of: automation potential, high throughput analysis of large quantities of samples, high reliability due to reduced human intervention, miniaturization potential on the size of analytical cell/device, near-/real-time detection, integration with other analytical systems and capability for the simultaneous detection of As(III), Cd(II), and Pb(II). Additionally, the flow cell geometry was optimized and finally confirmed by COMSOL Multiphysics to resolve the problems existing in the application of SPEs in flow system based on a finite element method (FEM), which is a reliable and effective approach for the computational fluid dynamic (CFD) analysis. Furthermore, the polymer ASV flow cell with desired geometry and materials was fabricated by 3D-printing. Moreover, highly flexible SPEs were prepared by screen-printing a commercial graphite ink and an Ag/AgCl ink onto polyimide substrate. The SPEs were composed of two WEs, a CE and a Ag/AgCl quasi-RE. As well, two sensing nanocomposites, i.e., (BiO)_2_CO_3_-reduced graphene oxide (rGO)-Nafion ((BiO)_2_CO_3_-rGO-Nafion) and Fe_3_O_4_ magnetic nanoparticles (Fe_3_O_4_MNPs) @Au nanoparticles (AuNPs)-ionic liquid (IL) (Fe_3_O_4_-Au-IL), with different catalytic properties were used to modify the two WEs integrated in SPE separately based on our previous works (Zhao et al., 2020; Sedki et al., 2021) with small changes to obtain a good sensitivity and selectivity, as compared with the bare SPEs. On this basis, a homemade flow system for the stripping voltammetry analysis of HMIs was developed, comprising a nanocomposites-modified SPE integrated with an 3D-printed flow cell. The proposed flow injection system exhibited satisfactory results for the detection and quantification of Pb(Ⅱ), As(Ⅲ), and Cd(Ⅱ) in simulated river water samples.

## Experimental

### Reagents and Instruments

Cd(II) and Pb(II) standard solutions were obtained from Sigma-Aldrich (USA) and diluted as required before using. Arsenic trioxide (As_2_O_3_) was purchased from Strem Chemicals, INC. (USA). An As(III) stock solution (1 mg/ml) was prepared by dissolving As_2_O_3_ in the 1.0 M aqueous NaOH. Carbon Graphite Paste (C2030519P4) was purchased from Gwent Group (UK) and Silver Silver/Chloride Ink (Electrodag 7019) was purchased from Tekra (USA). Polyimide film (5 mil) was purchased from Amazon.com (USA). Sodium acetate trihydrate was obtained from Fisher Scientific (USA) and prepared as acetate buffer solution (0.2 M) with acetic acid for the electroanalysis of As(III), Pb(II), and Cd(II). All the above chemicals and reagents were analytically pure.

Scanning electron microscopy (SEM) (NovaNanoSEM 450) was used for surface characterization of the sensing nanocomposite modified SPE. A peristaltic pump was used to construct the sequential injection system that was obtained from Rainin Instrument Co., Inc. (CA, USA). Squeegee and screen mesh were obtained from Rheeliable Screen Print Supply (CA, USA). Square wave stripping voltammetry (SWASV) was performed on a CH Instrument 760C electrochemical work station using the proposed SPEs. Electrochemical impedance spectroscopy (EIS) and cyclic voltammetry (CV) were performed on the above electrochemical work station with a three-electrode system consisting of an Ag/AgCl reference electrode, a Pt wire counter electrode and a working electrode, i.e., GCE or the working electrode of SPE. The diameter of both the working electrodes was 3 mm. An optical microscope (KH-7700, Hirox, Japan) was used to characterize the thickness of the ink layers and a 3D printer Form2 (FormLabs, Somerville, MA, United States) was used to fabricate the flow cell.

### Design and Preparation of SPE

**FIGURE 1:**
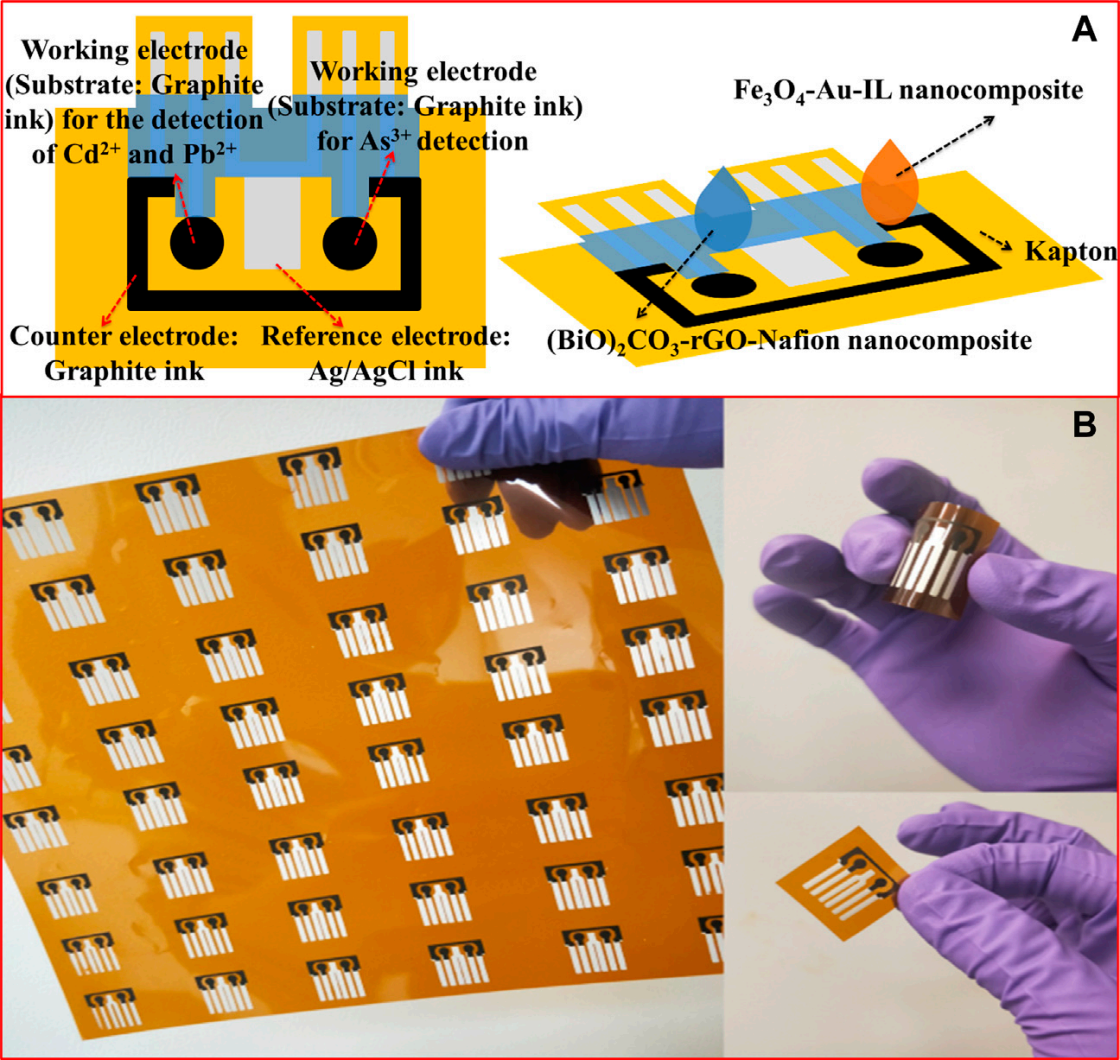
**(A)** Schematic diagram of the SPE. **(B)** Optical images of the SPE.

To meet the requirements of simultaneous detection of Pb(II), Cd(II), and As(III), a flexible SPE configured with two WEs, one CE, and one RE was designed and fabricated. Additionally, the polyimide film was used as the substrate of the SPE. The WE and CE were made of graphite ink while Ag/AgCl ink was used for the preparation of the Ag/AgCl quasi-RE, as shown in Figure 1A. Moreover, two kind of sensing nanocomposites, i.e., (BiO)_2_CO_3_-rGO-Nafion and Fe_3_O_4_-Au-IL were drop casted on the two working electrodes, where (BiO)_2_CO_3_-rGO-Nafion was used for the detection of Pb(II) and Cd(II) and Fe_3_O_4_-Au-IL was used for the detection As(III). As shown in Figure 1B, the proposed SPE with a flexible polyimide substrate is flexible and amenable to incorporation in flow cell.

The procedure of SPE fabrication based on screen-printing is shown in Supplementary Figure S1, which illustrates the printing process of graphite ink and Ag/AgCl ink. The Ag/AgCl ink and graphite ink were printed separately on the polyimide substrate by the sweeping of a rubber squeegee across the screen surface. The desirable electrodes were obtained while the squeegee swept over an area on polyimide substrate, depositing the ink onto the substrate surface. After that, the obtained SPEs were dried in the oven at 60°C for 1 h. The patterning of the desirable electrode printed on the polyimide substrate was achieved by controlling the area with holes on the screen template, i.e., electrode pattern.

### Fabrication of Flow Cell

The flow cell was first designed using Autodesk Inventor (Autodesk, San Rafael, CA, United States), and 3D-printed using a Form2 printer (FormLabs, Somerville, MA, United States), as shown in Figure 5. The flow cell was fabricated *via* stereolithography using standard clear resin from FormLabs. After that, the prepared flow device was washed in an isopropanol sonication bath for 20 min. The device was blown dry with pressurized air and photocured for 1.5 h at 60°C under ultraviolet light. The flow cell consists of two parts, i.e., bottom part and top part, which were assembled together using screws and nuts, as shown in Supplementary Figure S2. A sealed flow channel was formed by a gasket between top part and bottom part. The inlet and outlet were installed on the top of the flow cell, and a support was designed and used as an upholder for SPE.

### Synthesis and Modification of Sensing Nanocomposites

The (BiO)_2_CO_3_-rGO-Nafion and Fe_3_O_4_-Au-IL sensing nanocomposites were synthesized based on previously reported protocols (Zhao et al., 2020; Sedki et al., 2021) using the following steps.

(BiO)_2_CO_3_-rGO-Nafion sensing nanocomposite: Briefly, a 60 ml mixture of 5 mg/ml Bi(NO_3_)_3_.5H_2_O and 0.5 mg/ml graphene oxide (GO) was prepared to obtain the GO-Bi^3+^ mixed solution. Then the mixed solution was chemically reduced by adding 0.51 g NaBH_4_ at 60°C with continuous stirring for 2 h. The (BiO)_2_CO_3_-rGO deposit was obtained by the centrifuging of the above solution. Subsequently, the obtained deposit was washed for three times to remove the solvent residues, and then the deposit was placed in the oven at 50°C overnight to totally dry. 1 mg of obtained solid composite was added into 4 ml DMF and sonicated for 20 min. After that, 0.5 wt% Nafion solution with a volume of 800 μL was mixed with the above 4 ml (BiO)_2_CO_3_-rGO solution to get the (BiO)_2_CO_3_-rGO-Nafion suspension.

Fe_3_O_4_-Au-IL sensing nanocomposite: A 40 ml mixed solution of 0.8 mg/L FeCl_3_·6H_2_O and 0.5 mg/L FeCl_2_·4H_2_O was prepared using ethylene glycol (EG) as solvent. Next, the above solution was heated to 80°C and 0.15 ml PVA (1%) was added to the mixture. Then the pH of the solution was adjusted to 10.0 by dropwise addition of ammonium hydroxide (5% v/v). The liquid-solid separation of the obtained mixture was achieved by applying an external magnetic field, and then the separated solid composite was washed several times with DI water to remove the solvent. The resulting black product was dried at 60°C under vacuum for 24 h. The decoration of Fe_3_O_4_MNPs on AuNPs was accomplished by adding 5 mg Fe_3_O_4_ to 10 ml of 2 mg/ml HAuCl_4_·3H_2_O aqueous solution with a sonication. Then 10 ml sodium citrate (1.0%) was added to the solution of Fe_3_O_4_-Au^3+^ at 80°C under vigorous stirring for an hour to obtain the AuNPs-Fe_3_O_4_. After that, 100 μL 0.5% ionic liquid was mixed with the above solution to obtain the Fe_3_O_4_-Au-IL suspension.

Then 6 μL of a (BiO)_2_CO_3_-rGO-Nafion suspension and 6 μL of a Fe_3_O_4_-Au-IL suspension were modified on the two working electrodes integrated in SPE, separately, and solidified in oven at a temperature of 60°C.

### Preparation of the Simulated River Water Samples

Water samples simulating the composition of river water spiked with varying concentrations of HMIs were prepared to evaluate the analytical performance of the proposed detection system. The simulated river water consisted magnesium nitrate (150 ppm), ammonium chloride (60 ppm), potassium chloride (500 ppm), sodium citrate (50 ppm) and calcium chloride (500 ppm). The concentration of total dissolved solids (TDS) was 1,260 ppm. The analyte solution consisting of simulated water and acetate buffer (2.0 M, pH 5.0) with a volume ratio of 9:1 was used for each measurement to guarantee the pH 5.0 buffer condition with 0.2 M acetate.

### Analysis Procedure of the Flow Injection System

**FIGURE 2:**
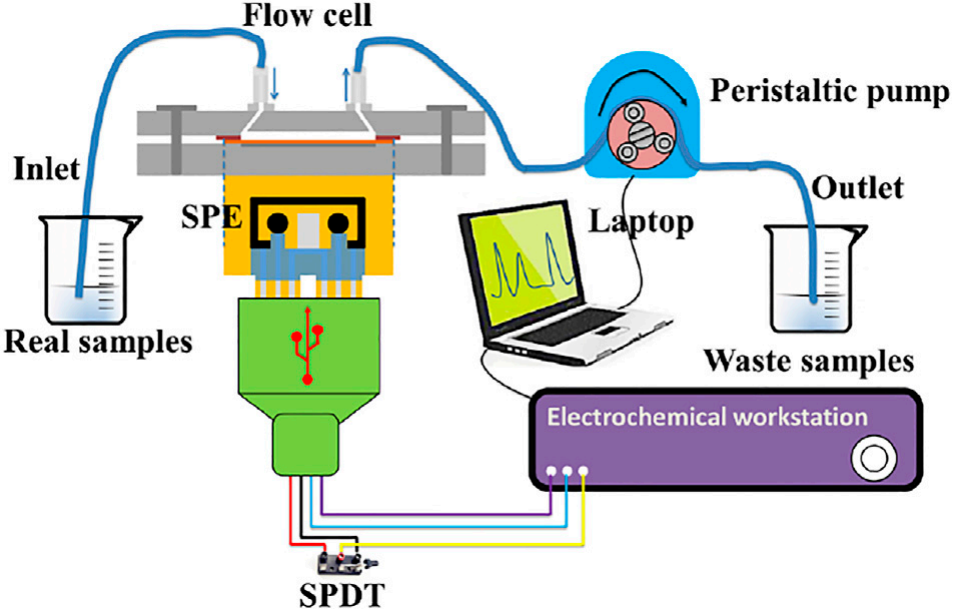
Schematic illustration of the flow injection system for the analysis of HMIs.

The setup of the flow injection system is illustrated in Figure 2. A peristaltic pump was used to deliver the samples mixed with acetate buffer (pH 5.0, 0.2 M) through the tube and flow cell at a flow rate of 120 μL/s. The deposition and stripping steps were carried out using an electrochemical workstation that was connected to the SPE. The injected standards used to establish calibration plots were composed of acetate buffer (0.2 M, pH 5.0) with different concentrations of Cd(Ⅱ), Pb(Ⅱ), and As(Ⅲ). The SWASV detection was carried out after the baseline signal of WEs in fluid channel has stabilized.

The flow injection analysis procedure should be consistent with the detection procedure of SWASV, which can be divided into three steps. In the first step, the water sample in the beaker was delivered to the flow cell through the channel by the pumping while applying the deposition (metal ions were electrochemically reduced to metal) potential of −1.2 V for 200 s on the Cd(Ⅱ) and Pb(Ⅱ) WE and −0.9 V for 250 s on the As(Ⅲ) WE. During step 2, the pumping was manually paused first for 10 s, and a potential scanning (frequency, 25 Hz; increment potential, 5 mV; amplitude, 25 mV) was performed on WEs in a quiescent sample solution over a range of −0.55 to 0.05 V for As(Ⅲ) and −1.25 to −0.65 V for Pb(Ⅱ) and Cd(Ⅱ). Finally, the surfaces of WEs were activated/regenerated by applying a potential of −0.4 V for Cd(Ⅱ) and Pb(Ⅱ) and +0.6 V for As(Ⅲ) to remove the residual metals (step 3), which prepared the electrodes for a new cycle of stripping voltammetry analysis. The duration of each measurement was around 334.8 s for Cd(Ⅱ) and Pb(Ⅱ) and ∼384.8 s for As(Ⅲ). All the measurements were carried out in 0.1 M acetate buffer (pH = 5.0). A single-pole double-throw switch was used to electrically isolate the specific working electrode’s electrochemical detection and quantification of the corresponding target analyte by switching the electrochemical workstation to connect with different working electrodes of SPE to achieve the detection of target HMIs serially.

### CFD Simulation Based on FEM

**FIGURE 3:**
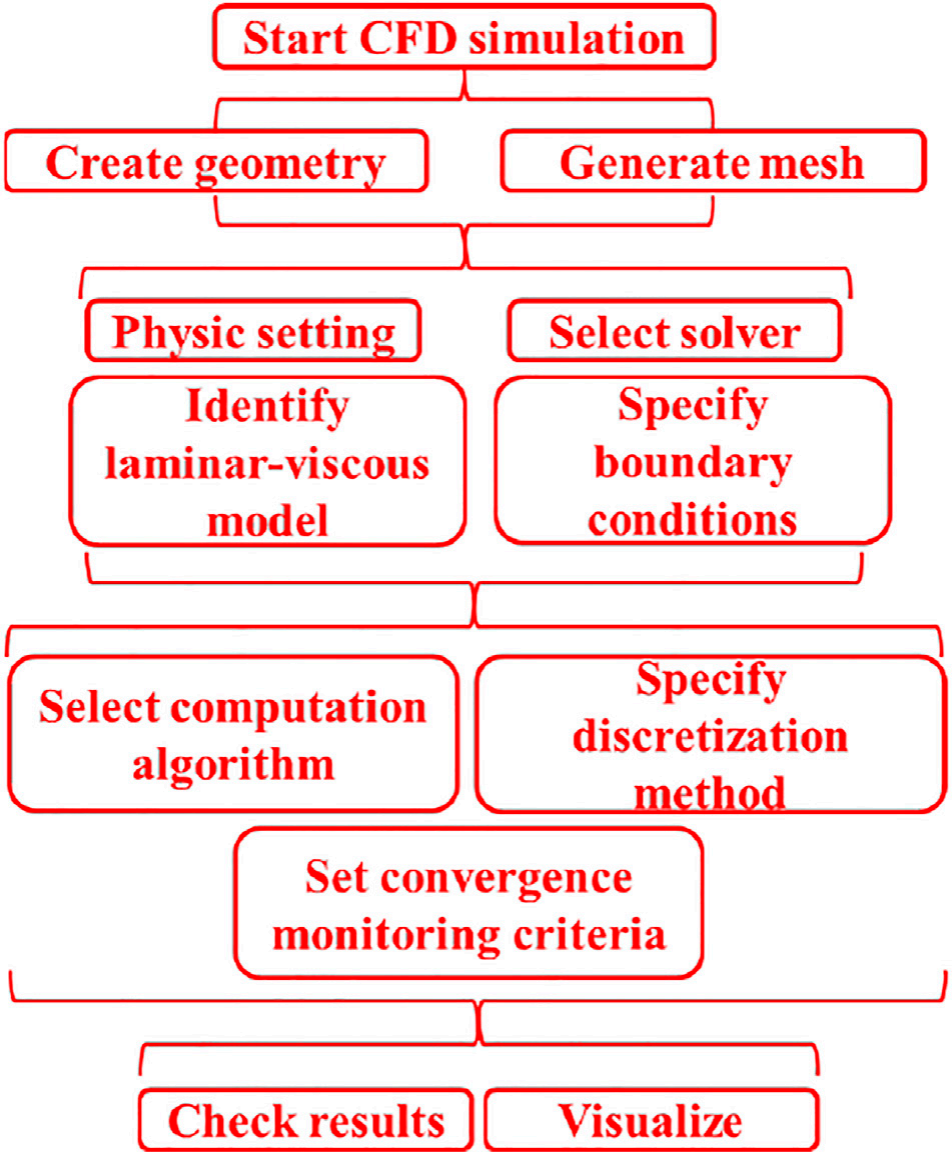
Flow chart of CFD numerical methodology.

CFD simulation based on FEM was completed based on the steps illustrated on flowchart in Figure 3 for the optimization of the desired structure of the flow electrochemical cell. In presented simulations non-compressible model of fluid was used, the fluid parameters, i.e., dynamic viscosity and density, were 0.89 cP and 1,000 kg/m^3^, respectively. Additionally, the boundary conditions were as follows: fluid inlet velocity equal to 0.012 m/s, fluid mass-flow equal to 400 μL/min, fluid velocity equal to 0 m/s on channel walls.

## Results and Discussion

### Optical Microscope Analysis of the Proposed SPE

The morphology of graphite layer and Ag/AgCl printed on polyimide was investigated by optical microscopy (Supplementary Figure S3). The images show the graphite ink formed a homogeneous and dense printing layer on the substrate (Supplementary Figure S3A) and Ag/AgCl layer had the grains in different sizes, which should be the AgCl and Ag, respectively, distributed uniformly on the substrate (Supplementary Figure S3B). It can be seen from the Supplementary Figures S3C,D, respectively, that the graphite layer and Ag/AgCl layer were printed stably on the polyimide substrate, and the boundary between carbon graphite layer and polyimide and the boundary between the Ag/AgCl layer and polyimide was clear and regular as well, demonstrating that the inks printed on the substrate were well distributed and controllable.

Supplementary Figure S4 shows the thickness of carbon graphite and Ag/AgCl layers printed on the polyimide substrate. The thickness of Ag/AgCl layer, used as RE, was 7.3 μm (Supplementary Figure S4A) and the thickness of carbon graphite layer, used as WE and CE, was 7.4 μm (Supplementary Figure S4B). Additionally, the carbon graphite layer printed on the Ag/AgCl layer can be seen in Supplementary Figure S4C, where Ag/AgCl layer was used as a conducting layer in this case. The results from the Supplementary Figure S4C indicated that the thickness of carbon graphite layer and Ag/AgCl layer were 7.2 and 7.3 μm, respectively, that were consistent with the results shown in Supplementary Figures S4A,B. This validated that the printing strategy used for the preparation of SPE in this study is reliable. Moreover, we investigated the thickness of graphite layer using simulation (Supplementary Figures S4D,E). The simulation of the graphite layer’s thickness was carried out using the built-in software of the optical microscope (KH-7700, 156 Hirox, Japan). As illustrated in Supplementary Figure S4E, the simulated thickness of the graphite layer was 8.473 μm, which was almost the same to the thickness determined from Supplementary Figure S4B. Additionally, the simulated results also indicated that the printing layer thickness is relatively uniform.

### Characterization of Sensing Nanocomposites Modified SPE

Electron microscopy, X-Ray Diffraction and Fourier transform infra-red spectroscopy were used to ensure the synthesized (BiO)_2_CO_3_-rGO and Fe_3_O_4_-Au nanocomposites had desired morphologies/structures and chemical compositions as reported in our previously published papers (Zhao et al., 2020; Sedki et al., 2021).

**FIGURE 4:**
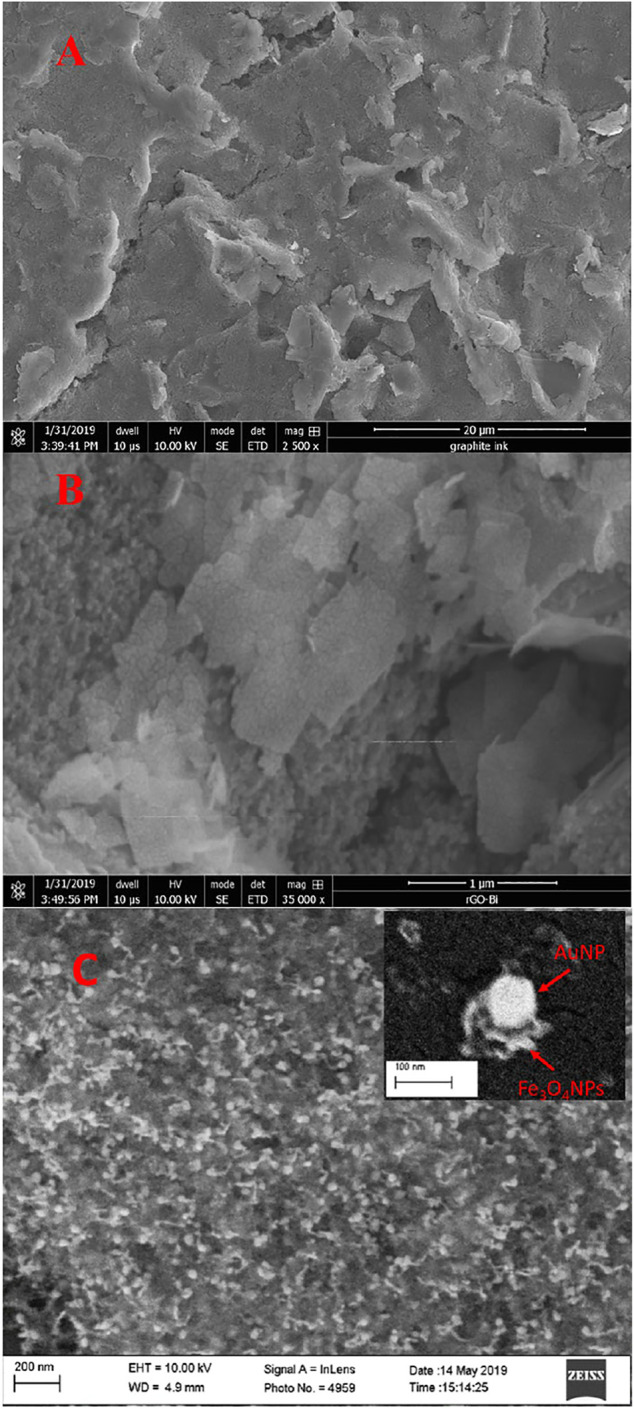
SEM images of **(A)** bare SPE, **(B)** (BiO)_2_CO_3_-rGO-Nafion/SPE and **(C)** Fe_3_O_4_-Au-IL/SPE.

The surface morphologies of bare SPE, (BiO)_2_CO_3_-rGO-Nafion-modified SPE ((BiO)_2_CO_3_-rGO-Nafion/SPE) and Fe_3_O_4_-Au-IL-modified SPE (Fe_3_O_4_-Au-IL/SPE) were further examined in this paper. To investigate the morphology of sensing nanocomposites on the WEs of SPE, SEM was performed to observe the morphological changes resulting from the modification process, as shown in Figure 4. The bare/unmodified WE of SPE displayed a sheet-like structure, where the sheets can be ascribed to the graphite layers according to the composition of carbon ink (Figure 4A). After modification of the WE with the (BiO)_2_CO_3_-rGO-Nafion nanocomposite, the electrode morphology changed drastically, as evident by the abundance of nanoparticles observed on the graphite sheets, indicating the formation and uniform distribution of bismuth nanoparticles on the electrode (Figure 4B). The graphite sheet as a substrate on the WE of SPE supply a large specific area (Zhao et al., 2017) for the modification of (BiO)_2_CO_3_-rGO-Nafion film. Similarly, the altered morphology of Fe_3_O_4_-Au-IL-modified WE of SPE can be observed in Figure 4C. Many nanoparticles were distributed on the electrode surface, which are attributed to AuNPs and Fe_3_O_4_MNPs. In addition, it can be seen from Figure 4C, inset, that the AuNP is covered with Fe_3_O_4_MNPs, which can be expected to obtain a high catalytic performance for As(Ⅲ) detection. Au and Fe_3_O_4_ NPs were identified from SEM and EDS, and EDS mapping results are shown in Supplementary Figure S5, where the color distribution of the elements elucidates their homogeneous distribution on the electrode surface. The catalytic activity of Fe_3_O_4_-Au-IL nanocomposite on the enhancement of SWASV response for As(Ⅲ) detection was investigated by our previous works (Zhao and Liu, 2019; Sedki et al., 2021).

**FIGURE 5:**
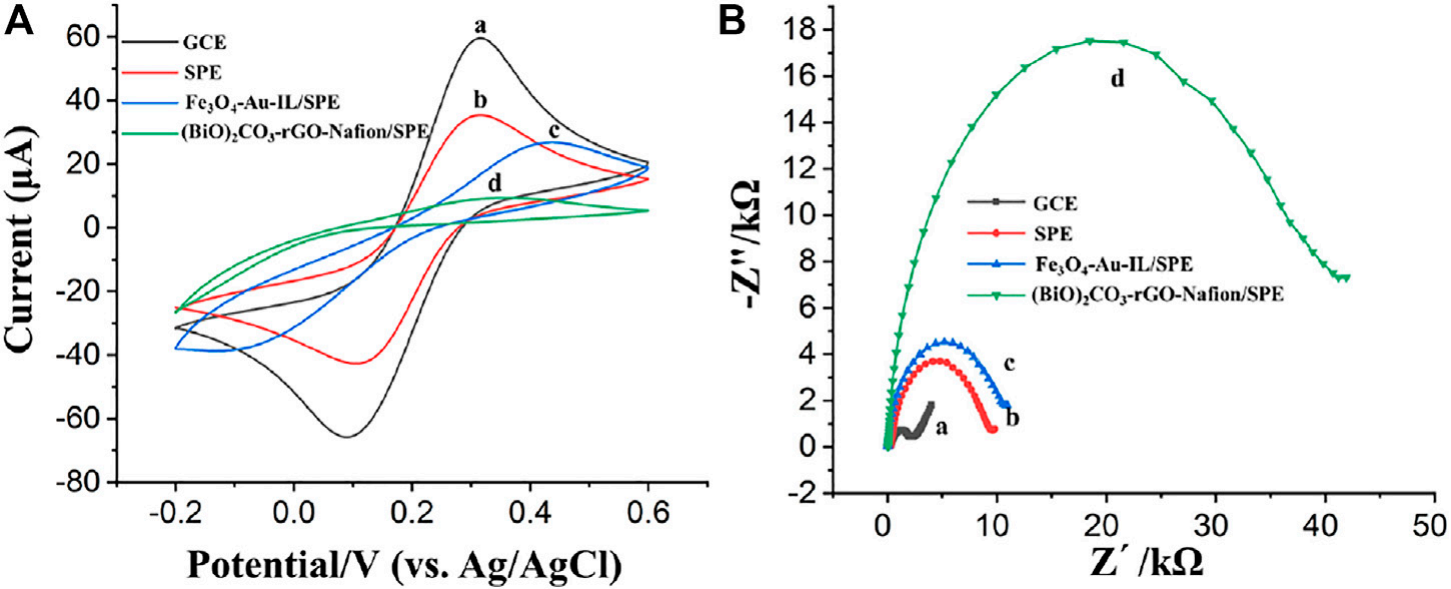
**(A)** Cyclic voltammograms of (a) GCE, (b) SPE, (c) Fe_3_O_4_-Au-IL/SPE, and (d) (BiO)_2_CO_3_-rGO-Nafion/SPE. **(B)** Electrochemical impedance spectroscopy (EIS) of (a) GCE, (b) SPE, (c) Fe_3_O_4_-Au-IL/SPE, and (d) (BiO)_2_CO_3_-rGO-Nafion/SPE. CV and EIS were performed with a three-electrode system consists of an Ag/AgCl reference electrode, a Pt wire counter electrode and a working electrode, i.e., GCE or the working electrode of SPE. The diameter of both the working electrodes was 3 mm.

The electrochemical behavior of bare WE of SPE, different nanocomposites modified WE of SPE and bare GCE was investigated and compared by cyclic voltammetry (CV) using a platinum wire and a Ag/AgCl electrode as CE and RE, respectively (Zhao et al., 2016). Figures 5Aa,Ab show the comparison of cyclic voltammograms obtained at a SPE and at a glassy carbon electrode (GCE) in an electrolyte with 0.1 M KCl and 5 mM [Fe(CN)_6_]^3−/4−^. As illustrated, well-defined anodic and cathodic peaks corresponding to the oxidation and reduction of the [Fe(CN)_6_]^3−/4−^ redox probe were obtained at bare GCE, which was larger compared with the peak currents obtained at bare SPE WE. This maybe because the multilayer graphite nanosheets hinder the electron transfer from one graphite sheet to another one and/or the binder, dispersant, and solvent used for the preparation of SPE could block the electron transfer. After the modification of Fe_3_O_4_-Au-IL nanocomposite and (BiO)_2_CO_3_-rGO-Nafion nanocomposite on the SPEs, respectively, both of the peak currents at the electrode surface decreased due to the significant negative effect of poor conductivity of Fe_3_O_4_ and Nafion on the electron transfer on the surface of SPE, which was consistent with our previous reported results (Zhao et al., 2020; Sedki et al., 2021).

Electrochemical impedance spectroscopy (EIS) was also used to further characterize the properties of the bare SPE, Fe_3_O_4_-Au-IL/SPE and (BiO)_2_CO_3_-rGO-Nafion/SPE, in which the charge transfer resistance (Rct) at the interface of electrode/electrolyte can be qualitatively evaluated by the semicircle of Nyquist plot (Jiang et al., 2013). The EIS was carried out on the electrochemical work station with a potential and amplitude of 0.25 and 0.01 V, respectively, over a frequency range of 0.1 Hz–10^5^ Hz. As shown in Figures 5Ba,5Bb, the semicircle diameter of bare SPE is larger than bare GCE, which indicated a large charge transfer resistance at the interface between electrode and electrolyte composed of 0.1 M KCl and 5 mM [Fe(CN)_6_]^3−/4−^. The EIS analysis of Fe_3_O_4_-Au-IL/SPE and (BiO)_2_CO_3_-rGO-Nafion/SPE can be seen in Figures 5Bc,Bd, respectively, which pointing out an increasing trend compared with bare SPE. A possible reason for this phenomenon can be ascribed to the poor electrical conductivity of Fe_3_O_4_ and Nafion. The analysis results of EIS were consistent with those of CV and our previous investigation results as well (Zhao et al., 2020; Sedki et al., 2021).

### CFD Simulation of Flow Device Fabrication

**FIGURE 6:**
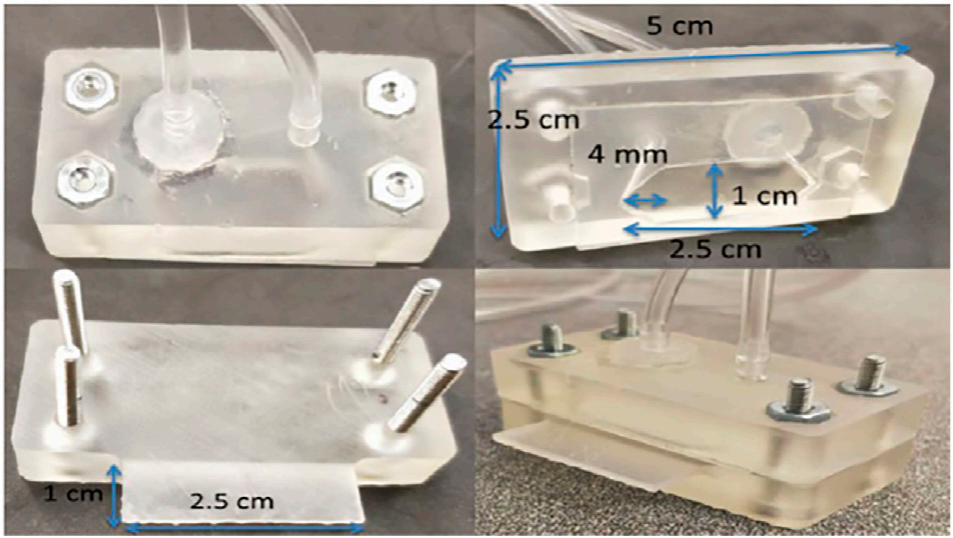
Optical images of the flow cell.

The computational fluid dynamic (CFD) simulation (Malecha and Golonka, 2006; Malecha et al., 2011) was carried out to optimize the structure of the flow cell using COMSOL Multiphysics, and consequently obtain the optimum efficiency of deposition and stripping for the stripping voltammetry detection of HMIs. An incompressible 2D Navier–Stokes (NS) flow model was applied in the simulation of the flow characteristics in the flow cell. The flow along the inside wall of the flow cell was considered as a no-slip condition. Additionally, the water was used as an example of fluid for the CFD simulation with a density of 1,000 kg/m^3^ and a dynamic viscosity of 0.89 cP (Vasudev et al., 2013). Figure 6 shows the picture of real flow cell with specific dimension. The actual size of the desirable 3D printed flow cell was ∼1 mm high, ∼1 cm wide and ∼2.5 cm long. According to the Eqs 1, 2, the calculated value of Reynolds number (*Re*) of the flow cell for a velocity of 0.012 m/s was around 24.27, which indicates that flow is laminar (Re < 2200).

$$Re=\frac{\rho uL}{\mu }$$ (1)

$$Dh=\frac{4A}{P}$$ (2)

where *μ*, *u* and *ρ* were the dynamic viscosity, velocity and density of the fluid, respectively. In addition, *L* is the characteristic linear dimension; *Dh* is the hydraulic diameter of the pipe; *A* is the cross-sectional area; and *P* is the wetted perimeter.

Taking the processes of electrodeposition and stripping of the HMIs and the characteristics of the fluid into account, the equations of convection-diffusion (3) and NS (4) were used here.

$$\frac{\partial c}{\partial t}=D\nabla^{2}c-u\cdot \nabla c$$ (3)

$$\text{ρ}\left(\frac{\partial u}{\partial t}+u\cdot \nabla u\right)=\;-\nabla p+\;\mu \nabla^{2}u+f$$ (4)

where *u*, *μ* and *ρ* are velocity, dynamic viscosity and density of the fluid, respectively. Moreover, *c* and *D* are the concentration and diffusion coefficient of the species, respectively. Additionally, *p* is the pressure and *f* is the external force.

**FIGURE 7:**
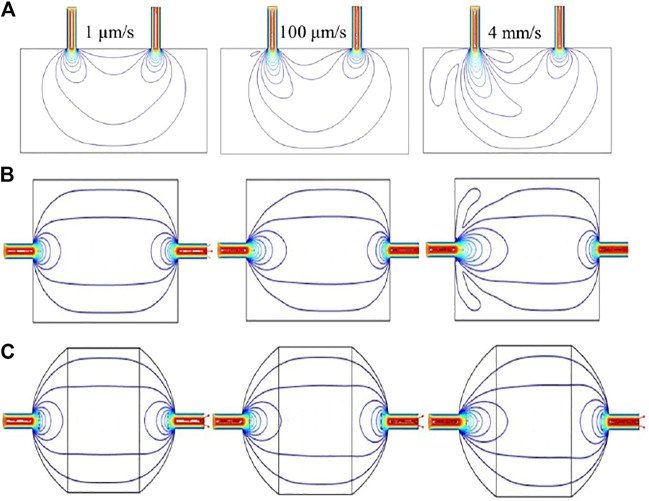
Results of CFD simulation in **(A)** square chamber (inlet and outlet are on the same side), **(B)** square chamber (inlet and outlet are on each side), and **(C)** elliptical chamber (inlet and outlet are on each side).

To begin, we investigated and optimized the effect of positioning the inlet and outlet on the top (same side) of the rectangular flow cell by the simulation of flow field at different fluid velocity, i.e. 1 μm/s, 100 μm/s and 4 mm/s, at the inlet with a no-slip boundary condition (Figure 7A). According the results of flow field simulation, small vortices were formed along with “dead zones,” i.e., regions without the distribution of flow, which can result in a negative effect on the deposition of HMs on the electrode. Moreover, when the fluid velocity increases to a high value, the samples solution will directly pass through the flow cell from inlet to outlet with a short residence time.

We next simulated the flow field trajectory in the flow cell with the inlet and outlet on opposite side of rectangle cell, as shown in Figure 7B. The results show that the flow field trajectories were uniformly distributed across the flow cell, but there were still some “dead zones.” Based on this simulation results, a modification for the previous design was carried out with a goal of enabling the shape of the flow cell match the observed elliptical flow field. The simulated flow field trajectories in the optimized flow cell with ellipse shape is shown in Figure 7C, which indicated that the “dead zones” was efficiently eliminated. Furthermore, a uniformly distributed flow field can be also found in the elliptic cell.

### Optimization of Detection Parameters

**FIGURE 8:**
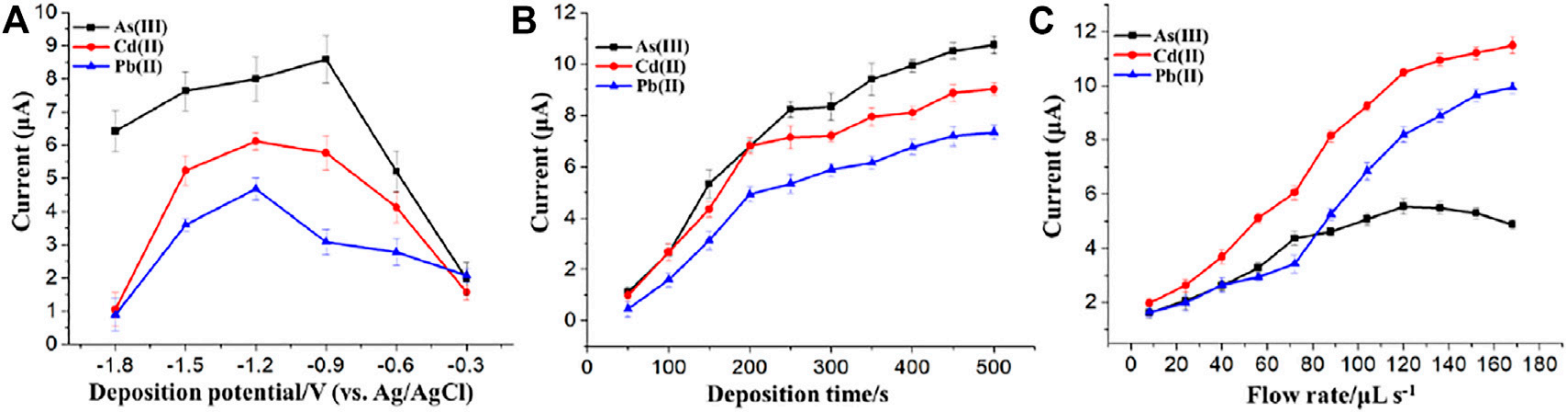
Effects of the **(A)** deposition potential, **(B)** deposition time and **(C)** flow rate on the stripping response of As(Ⅲ), Cd(II), and Pb(II). Each data point is an average of 3 measurements from 3 electrodes and error bars represent ±1 standard deviation.

To obtain a low detection limit and a high sensitivity, the effect of deposition potential and time on the stripping voltammetry response were examined using the flow system containing 50 μg/L As(Ⅲ) and 20 μg/L each of Pb(Ⅱ) and Cd(Ⅱ) in a wide range of parameter values. Deposition potential over a range of −0.3 V to −1.8 V was investigated, as shown in Figure 8A, where a highest stripping current can be found at the potential of −1.2 V for Pb(Ⅱ) and Cd(Ⅱ) and −0.9 V for As(Ⅲ). Therefore, deposition potentials of −0.9 V for As(Ⅲ) and −1.2 V for Cd(Ⅱ) and Pb(Ⅱ) were chosen for the following experiments.

The influence of deposition time on stripping voltammetry response of different modified electrodes is presented in Figure 8B. The stripping voltammetry responses of Pb(Ⅱ) and Cd(Ⅱ) and As(Ⅲ) were slightly changed after the deposition time reached a specific value from the range of 50–500 s. Taking both the efficiency and sensitivity into account, 200 and 250 s were selected as the deposition time and used for the stripping voltammetry detection of Pb(Ⅱ)/Cd(Ⅱ) and As(Ⅲ), respectively, in subsequent experiments.

As shown in Figure 8C, the optimal value of the delivery flow rate was investigated. For the flow injection analysis of 30 μg/L each of Pb(II) and Cd(II), the stripping peak currents rapidly increased when the flow rate increased from 8 to 120 μL/s and increased slightly from 120 to 168 μL/s. However, the stripping peak currents of 20 μg/L As(Ⅲ) began to decrease when the flow rate was above 120 μL/s. In order to reduce the consumption of the reagents and the samples and increase the preconcentration efficiency, 120 μL/s was selected as the flow rate for the flow injection analysis of the three HMIs. The reasons for the decrease of the stripping current of As(Ⅲ) as the flow rate increased above 120 μL/s can be explained as follows. The effect of flow rate on the stripping currents of HMIs was determined by the compromise between the thickness of diffusion layer and the time of residence. The time of residence at a lower flow rate was longer while the diffusion layer was thicker, which lead to a decrease of the As(Ⅲ) stripping voltammetry response. However, the time of residence was too short at a higher flow rate (Kokkinos et al., 2016), although the thickness of the diffusion layer decreased.

### Investigation of Electrode Precision

In order to verify the detection performance of the proposed electrodes, i.e., Fe_3_O_4_-Au-IL/SPE and (BiO)_2_CO_3_-rGO-Nafion/SPE, inter-electrode precision was investigated by the repetitive SWASV measurements of 20 μg/L Pb(II), Cd(II), and As(Ⅲ) using five different electrodes, and reproducible results can be observed with relative standard deviations (RSDs) of 4.53% for Cd(II), 5.54% for Pb(II), and 3.36% for As(Ⅲ). Additionally, intra-electrode precision of the proposed electrodes was further investigated by the five repeated SWASV measurements of 30 μg/L Pb(II), Cd(II), and As(Ⅲ) using one electrode, which the RSDs were 2.68%, 2.95%, and 2.28% for Cd(II), Pb(II), and As(Ⅲ), respectively. The proposed electrodes exhibited excellent inter-electrode and intra-electrode precision in repeated SWASV measurements of target metal ions under the optimal conditions. Moreover, the stability of the proposed electrodes was satisfying, as the stripping response of the proposed electrodes for Cd(II), Pb(II), and As (Ⅲ) hardly changed after keeping in air for 1 week, which meet the detection requirement of wastewater.

### Quantitative Detection of HMIs Using the Flow System

**FIGURE 9:**
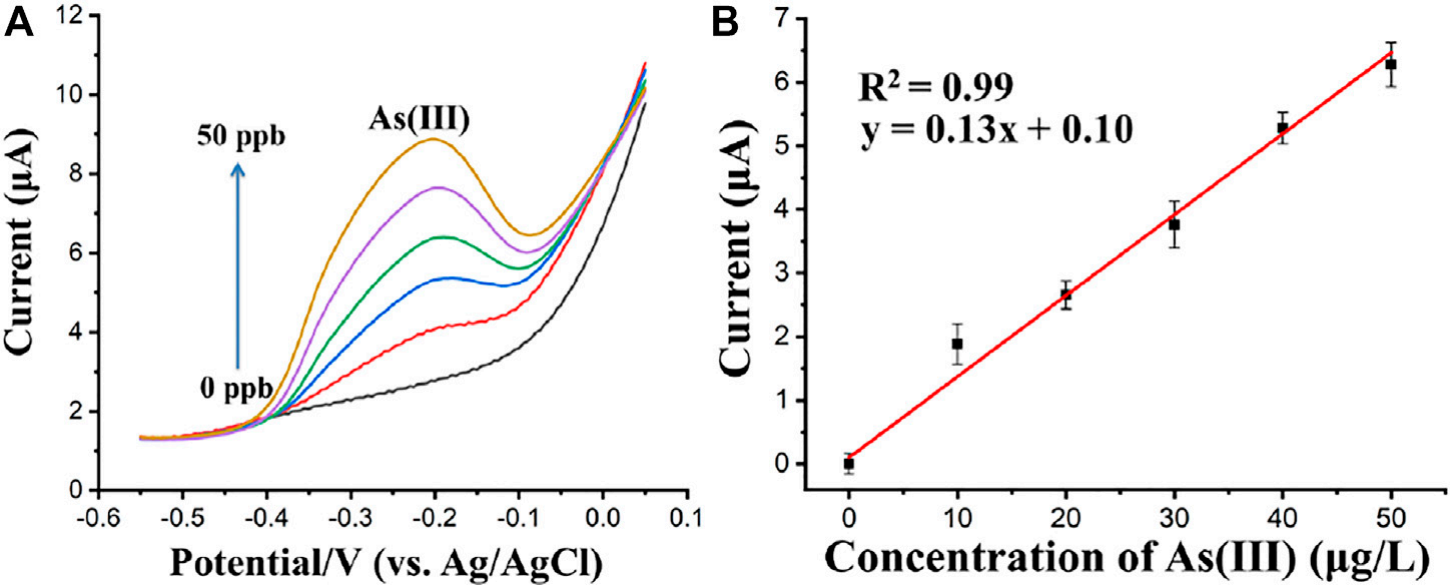
**(A)** Square wave anodic stripping voltammograms of As(Ⅲ) (0, 10, 20, 30, 40, and 50 μg/L). **(B)** The calibration curves for As(Ⅲ). Each data point is an average of 3 measurements from 3 electrodes and error bars represent ±1 standard deviation.

After the optimization of the SWASV parameters and flow rate, the flow system was applied in the detection of Cd(II), Pb(II), and As(Ⅲ) in acetate buffer solution. In the case of As(Ⅲ) detection, as shown in Figure 9A, Fe_3_O_4_-Au-IL modified electrode was used as WE with a deposition potential of −0.9 V for 250 s. The concentration of As(Ⅲ) was proportional to its corresponding stripping peak current over a concentration range from 0 μg/L to 50 μg/L based on the optimal parameters obtained above, in which the correlation coefficient and equation of the linear regression were *R* ^2^ = 0.99 and Peak current = 0.13*Concentration of As(Ⅲ) + 0.1, respectively, as shown in Figure 9B. The standard deviations of the slope and intercept of the linear regression equation were 0.005 and 0.133, respectively. Additionally, the limit of detection (LOD) of the Fe_3_O_4_-Au-IL modified electrode integrated in the proposed flow injection system for the stripping voltammetry analysis was 2.4 μg/L (S/N = 3). The LOD was calculated based on the equation of LOD = 3 × SD/S, in which SD is the standard deviation of blank response and S is the slope of calibration plot.

**FIGURE 10:**
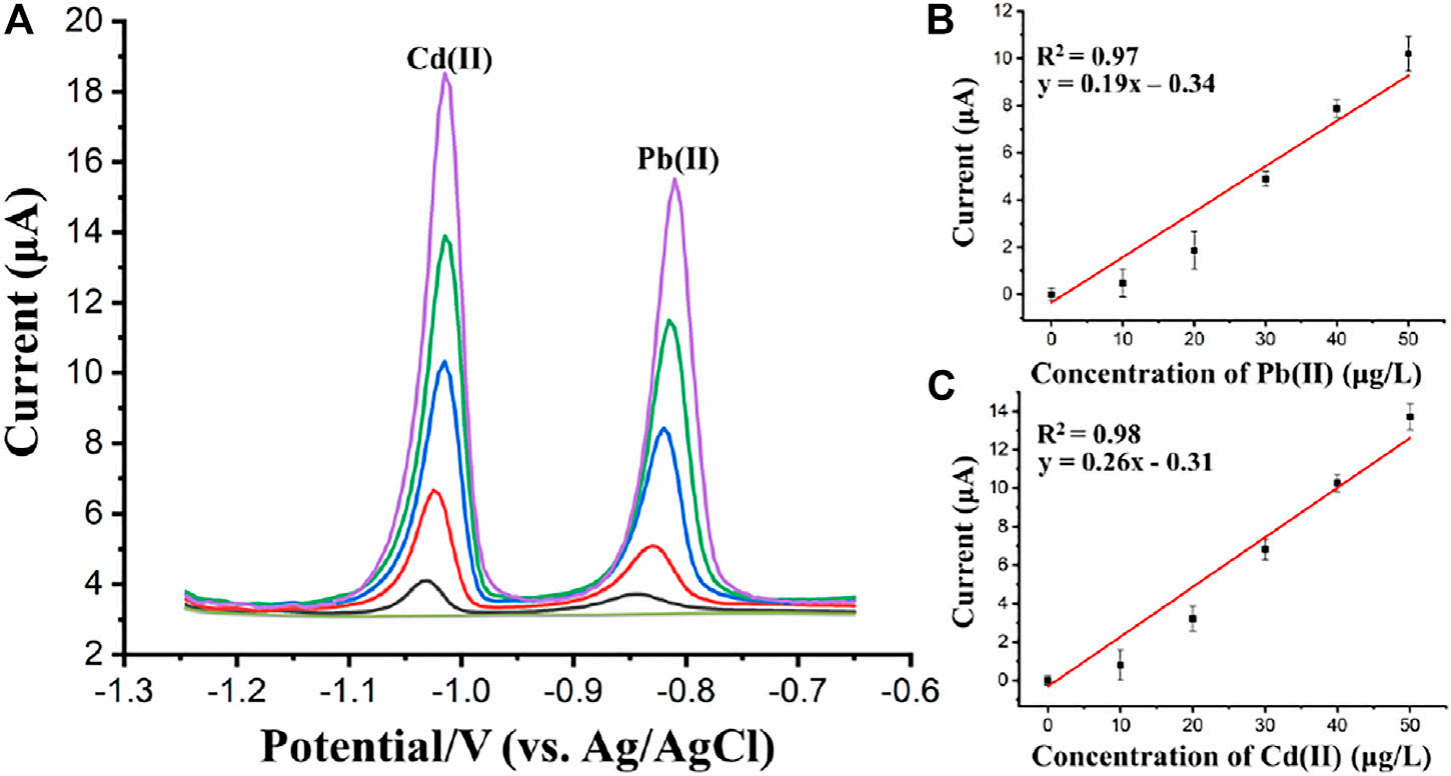
**(A)** Square wave anodic stripping voltammograms of Cd(II) and Pb(II) (0, 10, 20, 30, 40, and 50 μg/L). The calibration curves for Pb(II) **(B)**, and Cd(II) **(C)**. Each data point is an average of 3 measurements from 3 electrodes and error bars represent ±1 standard deviation.

The detection performance of the proposed flow system was also verified by the analysis of Cd(II) and Pb(II) using SWASV. Figure 10A presents SWASV curves for different concentrations of Pb(II) and Cd(II) obtained at (BiO)_2_CO_3_-rGO-Nafion/SPE in acetate buffer (pH 5.0). The measurements of the SWASV were carried out using a deposition potential of −1.2 V for 200 s with a potential scanning from −1.25 V to −0.65 V in the stripping step. The stripping voltammetry responses for Pb(II) and Cd(II) showed monotonic linear relationship with increasing concentrations over a range of 0–50 μg/L, which can be seen in the calibration plots with linear regression analyses, as shown in Figures 10B,C, respectively. The regression equations were obtained as, Peak current = 0.19*Concentration of Pb(II)−0.34 (*R* ^2^ = 0.97) for Pb(II); and Peak current = 0.26*Concentration of Cd(II)−0.31 for Cd(II). The standard deviations of the slope and intercept of the linear regression equations for Pb(II) and Cd(II) were 0.018 and 0.469, 0.020, and 0.473 respectively. The LODs were estimated to be 1.2 μg/L for Pb(II) and 0.8 μg/L for Cd(II), respectively. The interference of non-target ions, such as Mg^2+^, K^+^, Na^+^, Fe^2+^, Mn^2+^, Zn^2+^, Ca^2+^, Cu^2+^, Cl^−^, F^−^, NO_3_^−^, CO_3_^2−^, PO_4_^2−^ and SO_4_^2−^ at 10^2^-fold higher concentrations than As(III) and 10^4^-fold higher concentrations than Pb(II) and Cd(II) on the stripping responses of target HMIs was investigated. The results showed that, amongst the non-target ions, there was a small interference from Zn(II) on Pb(II) and Cd(II) detection at the (BiO)_2_CO_3_-rGO-Nafion/SPE. However, the concentration of Zn(II) in most waters is at trace level, which would not make a significant influence on the stripping current of Cd and Pb (Zhao et al., 2020; Sedki et al., 2021). Additionally, the results also showed peak current decrease of ∼28.56 and ∼24.92%, respectively, for Cd(II) and Pb(II) at the (BiO)_2_CO_3_-rGO-Nafion/SPE in presence of 10^4^ fold higher Cu(II) concentration and ∼18.95% for As(III) at the Fe_3_O_4_-Au-IL/SPE in presence of 10^2^ fold higher concentration of Cu(II). However, this would not make a significant influence on the stripping currents of target HMIs as the concentration of Cu(II) in most waters is at trace level. Furthermore, masking agent, such as hexacyanoferrate (II), that mask copper effectively without having a detrimental effect on the responses of target HMIs can be used to block Cu(II) interference (Crowley and Cassidy, 2002; Zhao et al., 2016). Furthermore, the detection performance comparison of several different reported flow devices are shown in Table 1. According to the results presented in Table 1, the proposed 3D-printed flow cell integrated with a nanocomposites modified SPE exhibited a comparable and even lower detection limit and less deposition time. Moreover, the proposed flow system was easy to fabricate with a low cost, and finally achieved the detection of Pb(II), Cd(II), and As(III) serially.

**TABLE 1:** Comparison of different flow devices used in the detection of HMs.

Flow device	Deposition time	HMs	LOD
Redha et al. (2009)	500 s/300 s	Cu(Ⅱ)/Pb(Ⅱ)	4.4 ppb/5.9 ppb
Hong et al. (2016)	180 s/180 s	Cd(Ⅱ)/Pb(Ⅱ)	0.5 ppb/0.2 ppb
Kokkinos et al. (2016)	114 s	Pb(Ⅱ)	0.5 ppb
Sun et al. (2017)	240 s	Pb(Ⅱ)	0.2 ppb
Henríquez et al. (2012)	200 s	Cd(Ⅱ)	0.79 ppb
This paper	200 s/250 s	Cd(Ⅱ) and Pb(Ⅱ)/As(Ⅲ)	0.8 and 1.2 ppb/2.4 ppb

### Application to Simulated River Water Samples

The stripping voltammetry analysis of Pb(II), Cd(II), and As(Ⅲ) in simulated/synthetic water samples was performed using the proposed flow system to estimate the applicability of the proposed platform composed of a 3D printed flow cell device coupled to a flexible SPE comprising two sensing nanocomposite modified working electrodes. The procedure of simulated river water experiment can be described as follows: First, the measurement was carried out for the initial water sample, and the corresponding results were obtained, i.e., the concentrations of water samples before spiking, which are listed in the second column of Table 2. After that, the standard solutions of specific HMIs were added into the water samples, consequently, the concentrations of water samples after spiking were obtained, i.e., the concentrations of water samples after spiking, which are listed in the fourth column of Table 2. As shown in Table 2, the average recoveries of Pb(II), Cd(II), and As(Ⅲ) were calculated to be 97.55%, 97.79%, and 97.98%, respectively, demonstrating excellent accuracy and selectivity of the sensor system for the targets even in presence of very high concentration of ions such as Mg^2+^, K^+^, Na^+^, NH_4_^+^ Ca^2+^, Cl^−^, NO_3_^−^ and citrate, and the feasibility/potential of the proposed system for environmental monitoring of Pb(II), Cd(II), and As(Ⅲ) in complex water samples.

**TABLE 2:** Results of multiplexed detection of Cd(II), Pb(II), and As(Ⅲ) in simulated water samples.

Sample no	The concentrations of water samples before spiking (μg/L)			Added (μg/L)	The concentrations of water samples after spiking (μg/L)			Recovery (%)		
	Cd(II)	Pb(II)	As(Ⅲ)		Cd(II)	Pb(II)	As(Ⅲ)	Cd(II)	Pb(II)	As(Ⅲ)
1	4.75	9.62	15.86	5	9.53	14.32	20.63	95.37	96.88	98.55
2	9.79	14.54	4.39	10	19.46	24.38	14.26	96.63	98.90	97.04
3	14.62	4.8	10.34	15	29.82	19.65	25.17	101.37	96.88	98.36

## Conclusion

In this paper, a flexible and disposable SPE for the stripping voltammetry measurement of Cd(II), Pb(II), and As(Ⅲ) coupled to a 3D-printed flow cell has been developed. The SPE integrated two different working electrodes modified by (BiO)_2_CO_3_-rGO-Nafion nanocomposite and Fe_3_O_4_-Au-IL nanocomposite for the SWASV determination of Cd(II)/Pb(II) and As(Ⅲ), respectively, which efficiently enhanced the sensitivity of the sensor. The design of the 3D printed flow cell was optimized by the CFD simulation to ensure efficient deposition of HMIs. The morphology and electrochemical property of different modified electrodes were also investigated. Additionally, experimental parameters for electrochemical detection of HMIs coupled with flow cell (e.g., flow rate, deposition period, and deposition potential) were optimized. The flow cell coupled with nanocomposite-modified multiplexed SPE provided effective and rapid detection of HMIs with low LODs and excellent recovery, which suggests a potential for future application in automated in-line detection of HMIs in the field.

## Data Availability

The original contributions presented in the study are included in the article/Supplementary Material, further inquiries can be directed to the corresponding author.

## References

[B1] Alpízar J. Cladera A. Cerdà V. Lastres E. García L. Catasús M. (1997). Simultaneous Flow Injection Analysis of Cadmium and lead with Differential Pulse Voltammetric Detection. Anal. Chim. Acta 340, 149–158. 10.1016/S0003-2670(96)00547-8

[B2] Ambrosi A. Moo J. G. S. Pumera M. (2016). Helical 3D-Printed Metal Electrodes as Custom-Shaped 3D Platform for Electrochemical Devices. Adv. Funct. Mater. 26, 698–703. 10.1002/adfm.201503902

[B3] Andrew Clayton T. Lindon J. C. Cloarec O. Antti H. Charuel C. Hanton G. (2006). Pharmaco-metabonomic Phenotyping and Personalized Drug Treatment. Nature 440, 1073–1077. 10.1038/nature04648 16625200

[B4] Au A. K. Huynh W. Horowitz L. F. Folch A. (2016). 3D-printed Microfluidics. Angew. Chem. Int. Ed. 55, 3862–3881. 10.1002/anie.201504382 PMC7679199 26854878

[B5] Becker H. Locascio L. E. (2002). Polymer Microfluidic Devices. Talanta 56, 267–287. 10.1016/S0039-9140(01)00594-X 18968500

[B6] Bi Z. Chapman C. S. Salaün P. van den Berg C. M. G. (2010). Determination of Lead and Cadmium in Sea- and Freshwater by Anodic Stripping Voltammetry with a Vibrating Bismuth Electrode. Electroanalysis 22, 2897–2907. 10.1002/elan.201000429

[B7] Chen C. Niu X. Chai Y. Zhao H. Lan M. (2013). Bismuth-based Porous Screen-Printed Carbon Electrode with Enhanced Sensitivity for Trace Heavy Metal Detection by Stripping Voltammetry. Sensors Actuators B Chem. 178, 339–342. 10.1016/j.snb.2012.12.109

[B8] Crowley K. Cassidy J. (2002). Trace Analysis of Lead at a Nafion-Modified Electrode Using Square‐Wave Anodic Stripping Voltammetry. Electroanalysis 14, 15–16. 10.1002/1521-4109(200208)14:15/16<1077:aid-elan1077>3.0.co;2-3

[B9] Davis A. C. Calloway C. P. Jr Jones B. T. (2007). Direct Determination of Cadmium in Urine by Tungsten-Coil Inductively Coupled Plasma Atomic Emission Spectrometry Using Palladium as a Permanent Modifier. Talanta 71, 1144–1149. 10.1016/j.talanta.2006.06.005 19071425

[B10] Economou A. (2010). Recent Developments in On-Line Electrochemical Stripping Analysis-An Overview of the Last 12 Years. Anal. Chim. Acta 683, 38–51. 10.1016/j.aca.2010.10.017 21094379

[B11] Erlenkötter A. Kottbus M. Chemnitius G. C. (2000). Flexible Amperometric Transducers for Biosensors Based on a Screen Printed Three Electrode System. J. Electroanal. Chem. 481, 82–94. 10.1016/s0022-0728(99)00491-x

[B12] Galani-Nikolakaki S. Kallithrakas-Kontos N. Katsanos A. A. (2002). Trace Element Analysis of Cretan Wines and Wine Products. Sci. Total Environ. 285, 155–163. 10.1016/S0048-9697(01)00912-3 11874038

[B13] Ge L. Yan J. Song X. Yan M. Ge S. Yu J. (2012). Three-dimensional Paper-Based Electrochemiluminescence Immunodevice for Multiplexed Measurement of Biomarkers and point-of-care Testing. Biomaterials 33, 1024–1031. 10.1016/j.biomaterials.2011.10.065 22074665

[B14] Gross B. C. Erkal J. L. Lockwood S. Y. Chen C. Spence D. M. (2014). Evaluation of 3D Printing and its Potential Impact on Biotechnology and the Chemical Sciences. Anal. Chem. 86, 3240–3253. 10.1021/ac403397r 24432804

[B15] Henríquez C. Laglera L. M. Alpizar M. J. Calvo J. Arduini F. Cerdà V. (2012). Cadmium Determination in Natural Water Samples with an Automatic Multisyringe Flow Injection System Coupled to a Flow-Through Screen Printed Electrode. Talanta 96, 140–146. 10.1016/j.talanta.2012.01.032 22817941

[B16] Hong Y. Wu M. Chen G. Dai Z. Zhang Y. Chen G. (2016). 3D Printed Microfluidic Device with Microporous Mn2O3-Modified Screen Printed Electrode for Real-Time Determination of Heavy Metal Ions. ACS Appl. Mater. Inter. 8 (48), 32940–32947. 10.1021/acsami.6b10464 27934187

[B17] Jiang Y.-Y. Wang K. Xu C.-Z. Yang X.-D. Li H.-H. (2013). Application of Alizarin/graphene-Chitosan Modified Electrode on Detection of Human Telomere DNA. Chin. J. Anal. Chem. 41, 481–487. 10.1016/S1872-2040(13)60641-6

[B18] Johnson B. N. Lancaster K. Z. Zhen G. He J. Gupta M. K. Kong Y. L. (2015). 3D Printed Anatomical Nerve Regeneration Pathways. Adv. Funct. Mater. 25, 6205–6217. 10.1002/adfm.201501760 26924958 PMC4765385

[B19] Kenawy I. M. M. Hafez M. A. H. Akl M. A. Lashein R. R. (2000). Determination by AAS of Some Trace Heavy Metal Lons in Some Natural and Biological Samples after Their Preconcentration Using Newly Chemically Modified Chloromethylated Polystyrene-PAN Ion-Exchanger. Anal. Sci. 16, 493–500. 10.2116/analsci.16.493

[B20] Kokkinos C. Economou A. Goddard N. G. Fielden P. R. Baldock S. J. (2016). Determination of Pb(II) by Sequential Injection/stripping Analysis at All-Plastic Electrochemical Fluidic Cells with Integrated Composite Electrodes. Talanta 153, 170–176. 10.1016/j.talanta.2016.03.025 27130105

[B21] Kolesky D. B. Truby R. L. Gladman A. S. Busbee T. A. Homan K. A. Lewis J. A. (2014). 3D Bioprinting of Vascularized, Heterogeneous Cell-Laden Tissue Constructs. Adv. Mater. 26, 3124–3130. 10.1002/adma.201305506 24550124

[B22] Leca B. Blum L. J. (2000). Luminol Electrochemiluminescence with Screen-Printed Electrodes for Low-Cost Disposable Oxidase-Based Optical Sensors. Analyst 125, 789–791. 10.1039/b002284p

[B23] Malecha K. Golonka L. J. (2006). “CFD Simulations of LTCC Based Microsystems,” in 2006 29th International Spring Seminar on Electronics Technology, St. Marienthal, Germany, 10–14 May, 2006 (IEEE), 156–160. 10.1109/ISSE.2006.365377

[B24] Malecha K. Pijanowska D. G. Golonka L. J. Kurek P. (2011). Low Temperature Co-fired Ceramic (LTCC)-based Biosensor for Continuous Glucose Monitoring. Sensors Actuators B Chem. 155, 923–929. 10.1016/j.snb.2011.01.002

[B25] Murphy S. V. Atala A. (2014). 3D Bioprinting of Tissues and Organs. Nat. Biotechnol. 32, 773–785. 10.1038/nbt.2958 25093879

[B26] Pandey S. K. Singh P. Singh J. Sachan S. Srivastava S. Singh S. K. (2016). Nanocarbon-based Electrochemical Detection of Heavy Metals. Electroanalysis 28, 2472–2488. 10.1002/elan.201600173

[B27] Panov V. P. Zykova I. V. Chekrenev S. A. (2008). Heavy Metals: The Industry and Environmental protection. Fibre Chem. 40, 241–245. 10.1007/s10692-008-9045-2

[B28] Puy-Llovera J. Pérez-Ràfols C. Serrano N. Díaz-Cruz J. M. Ariño C. Esteban M. (2017). Selenocystine Modified Screen-Printed Electrode as an Alternative Sensor for the Voltammetric Determination of Metal Ions. Talanta 175, 501–506. 10.1016/j.talanta.2017.07.089 28842024

[B29] Redha Z. M. Baldock S. J. Fielden P. R. Goddard N. J. Brown B. J. T. Haggett B. G. (2009). Hybrid Microfluidic Sensors Fabricated by Screen Printing and Injection Molding for Electrochemical and Electrochemiluminescence Detection. Electroanalysis 21 (3-5), 422–430. 10.1002/elan.200804415

[B30] Reyes D. R. Iossifidis D. Auroux P.-A. Manz A. (2002). Micro Total Analysis Systems. 1. Introduction, Theory, and Technology. Anal. Chem. 74, 2623–2636. 10.1021/ac0202435 12090653

[B31] Sedki M. Zhao G. Ma S. Jassby D. Mulchandani A. (2021). Linker-Free Magnetite-Decorated Gold Nanoparticles (Fe3O4-Au): Synthesis, Characterization, and Application for Electrochemical Detection of Arsenic (III). Sensors 21, 883. 10.3390/s21030883 33525604 PMC7866134

[B32] Silva E. L. Roldan P. d. S. Giné M. F. (2009). Simultaneous Preconcentration of Copper, Zinc, Cadmium, and Nickel in Water Samples by Cloud point Extraction Using 4-(2-Pyridylazo)-Resorcinol and Their Determination by Inductively Coupled Plasma Optic Emission Spectrometry. J. Hazard. Mater. 171, 1133–1138. 10.1016/j.jhazmat.2009.06.127 19646812

[B33] Sun Q. Wang J. Tang M. Huang L. Zhang Z. Liu C. (2017). A New Electrochemical System Based on a Flow-Field Shaped Solid Electrode and 3D-Printed Thin-Layer Flow Cell: Detection of Pb2+ Ions by Continuous Flow Accumulation Square-Wave Anodic Stripping Voltammetry. Anal. Chem. 89, 5024–5029. 10.1021/acs.analchem.7b00383 28393530

[B34] Tu J. Gan Y. Liang T. Wan H. Wang P. (2018). A Miniaturized Electrochemical System for High Sensitive Determination of Chromium(VI) by Screen-Printed Carbon Electrode with Gold Nanoparticles Modification. Sensors Actuators B Chem. 272, 582–588. 10.1016/j.snb.2018.06.006

[B35] Vasudev A. Kaushik A. Tomizawa Y. Norena N. Bhansali S. (2013). An LTCC-Based Microfluidic System for Label-free, Electrochemical Detection of Cortisol. Sensors Actuators B Chem. 182, 139–146. 10.1016/j.snb.2013.02.096

[B36] Verma N. Singh M. (2005). Biosensors for Heavy Metals. Biometals 18, 121–129. 10.1007/s10534-004-5787-3 15954738

[B37] Wan Z. Xu Z. Wang J. (2006). Flow Injection On-Line Solid Phase Extraction for Ultra-trace lead Screening with Hydride Generation Atomic Fluorescence Spectrometry. Analyst 131, 141–147. 10.1039/b511829h 16365675

[B38] West J. Becker M. Tombrink S. Manz A. (2008). Micro Total Analysis Systems: Latest Achievements. Anal. Chem. 80, 4403–4419. 10.1021/ac800680j 18498178

[B39] Whitesides G. M. (2006). The Origins and the Future of Microfluidics. Nature 442, 368–373. 10.1038/nature05058 16871203

[B40] Wu W. Wu P. Yang F. Sun D.-l. Zhang D.-X. Zhou Y.-K. (2018). Assessment of Heavy Metal Pollution and Human Health Risks in Urban Soils Around an Electronics Manufacturing Facility. Sci. Total Environ. 630, 53–61. 10.1016/j.scitotenv.2018.02.183 29475113

[B41] Wuana R. A. Okieimen F. E. (2011). Heavy Metals in Contaminated Soils: a Review of Sources, Chemistry, Risks and Best Available Strategies for Remediation. ISRN Ecol. 2011, 1–20. 10.5402/2011/402647

[B42] Zhao G. Liu G. (2019). Electrochemical Deposition of Gold Nanoparticles on Reduced Graphene Oxide by Fast Scan Cyclic Voltammetry for the Sensitive Determination of As(III). Nanomaterials 9, 41. 10.3390/nano9010041 PMC6359602 30597942

[B43] Zhao G. Yin Y. Wang H. Liu G. Wang Z. (2016). Sensitive Stripping Voltammetric Determination of Cd(II) and Pb(II) by a Bi/multi-Walled Carbon Nanotube-Emeraldine Base Polyaniline-Nafion Composite Modified Glassy Carbon Electrode. Electrochim. Acta 220, 267–275. 10.1016/j.electacta.2016.10.059

[B44] Zhao G. Wang H. Liu G. Wang Z. (2017). Simultaneous and Sensitive Detection of Cd(II) and Pb(II) Using a Novel Bismuth Film/Ordered Mesoporous Carbon-Molecular Wire Modified Graphite Carbon Paste Electrode. Electroanalysis 29, 497–505. 10.1002/elan.201600430

[B45] Zhao G. Sedki M. Ma S. Villarreal C. Mulchandani A. Jassby D. (2020). Bismuth Subcarbonate Decorated Reduced Graphene Oxide Nanocomposite for the Sensitive Stripping Voltammetry Analysis of Pb(II) and Cd(II) in Water. Sensors 20, 6085. 10.3390/s20216085 PMC7662973 33114759

