# Supplementary material for: Multiplexed Anodic Stripping Voltammetry Detection of Heavy Metals in Water Using Nanocomposites Modified Screen-Printed Electrodes Integrated With a 3D-Printed Flow Cell
Source: Front Chem. 2022 Feb 17;10:815805. doi: 10.3389/fchem.2022.815805 (PMC8892198; doi:10.3389/fchem.2022.815805)
Supplement: Supplementary file 1 [file DataSheet1.docx]

**Supplementary Information**

**Multiplexed Anodic Stripping Voltammetry Detection of Heavy Metals in Water Using Nanocomposites Modified Screen-Printed Electrodes integrated with a 3D-Printed Flow Cell**

Guo Zhao^1,2^, Thien-Toan Tran^3,†^, Sidharth Modha^3^, Mohammed Sedki^4^, Nosang V. Myung^1,†^, David Jassby^5^, Ashok Mulchandani^1,6,*^

^1^Department of Chemical and Environmental Engineering, University of California, Riverside, CA 92521, USA

^2^College of Artificial Intelligence, Nanjing Agricultural University, Nanjing, 210031, China

^3^Department of Bioengineering, University of California, Riverside, CA 92521, USA

^4^Materials Science and Engineering Program, University of California, Riverside, CA 92521, USA

^5^Department of Civil & Environmental Engineering, University of California, Los Angeles, CA, 90095, USA

^6^Center for Environmental Research and Technology (CE-CERT), University of California, Riverside, CA 92507, USA

^†^ Current address: Department of Chemical and Biomolecular Engineering, University of Notre Dame, IN 46556, USA

* Corresponding author, email: [adani@engr.ucr.edu](mailto:adani@engr.ucr.edu)


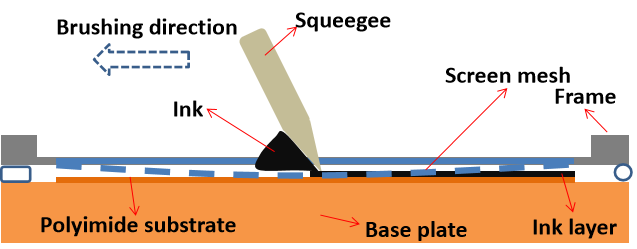


**Fig. S1.** Schematic diagram of the screen-printing process.


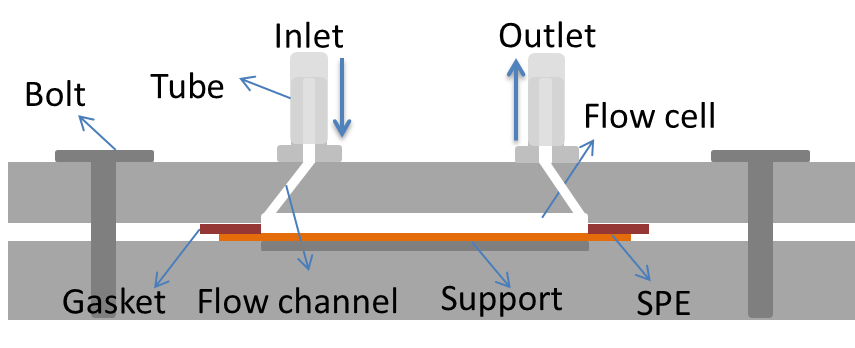


**Fig. S2.** Schematic diagram of the microfluidic cell.


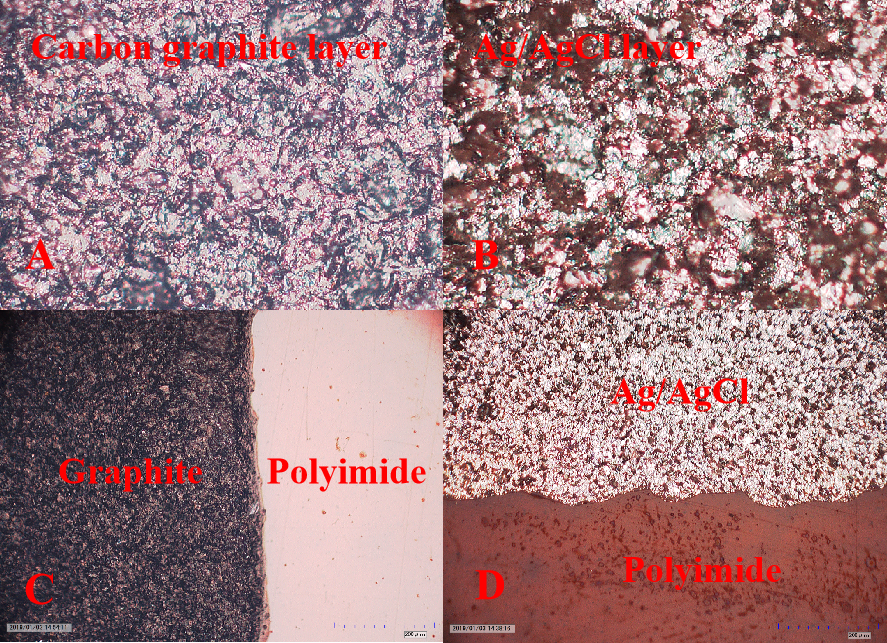


**Fig. S3.** Optical microscope images of (A) carbon graphite layer, (B) Ag/AgCl layer, (C) graphite layer/polyimide and (D) Ag/AgCl layer/polyimide.


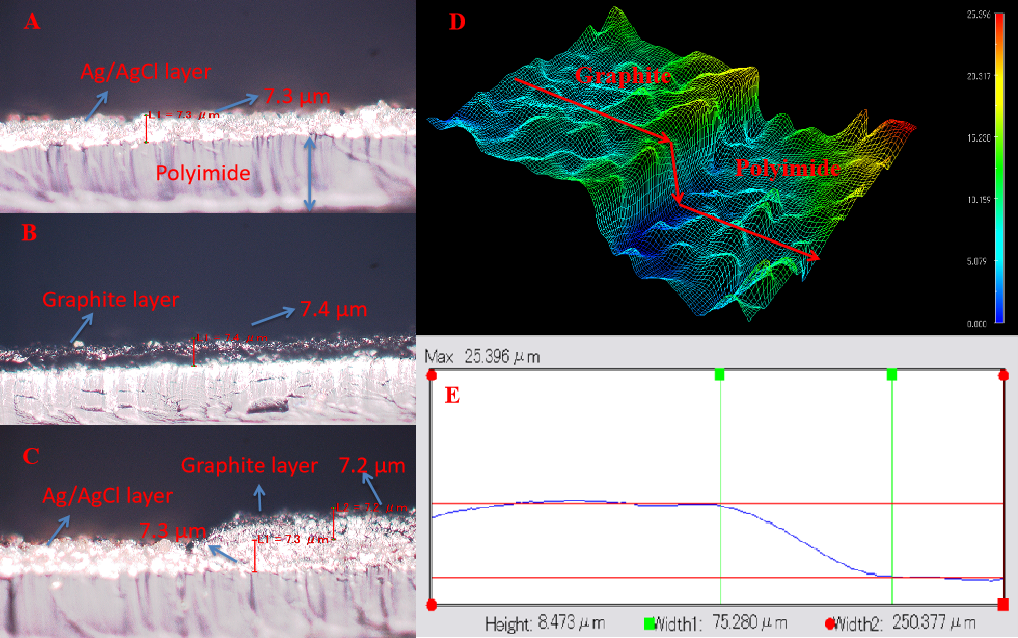


**Fig. S4.** Side view of the (A) Ag/AgCl layer, (B) graphite layer and (C) graphite layer printed on Ag/AgCl layer. (D) Simulation diagram of the morphology and (E) the simulation result of graphite layer thickness. Fig. S4 was created using the built-in software of the optical microscope (KH-7700, 156 Hirox, Japan).


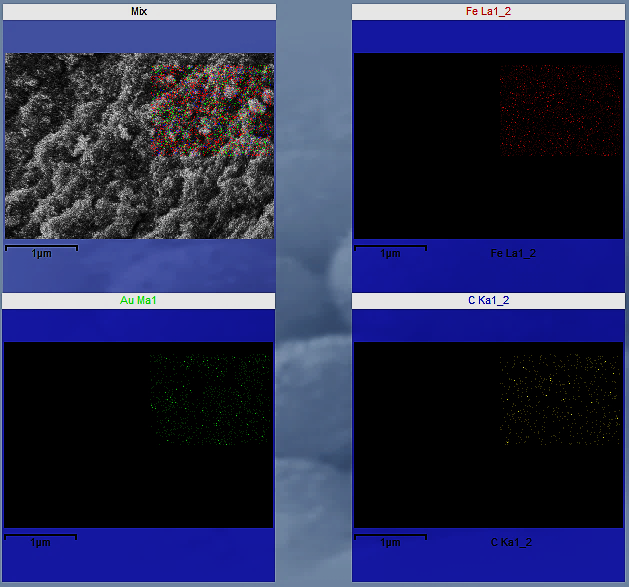

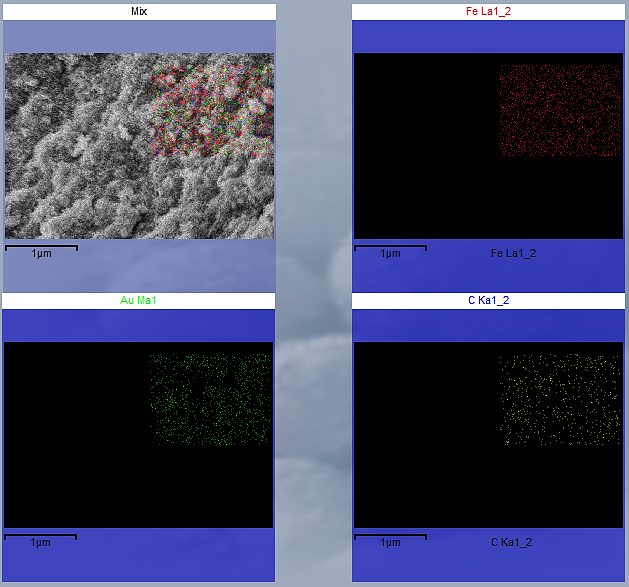

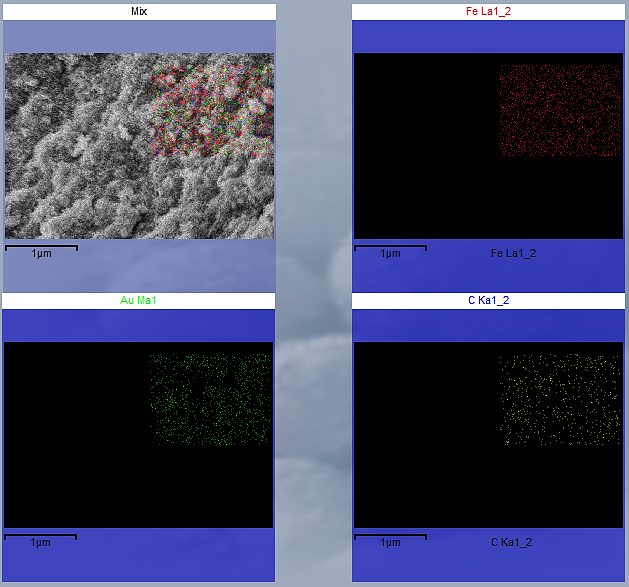

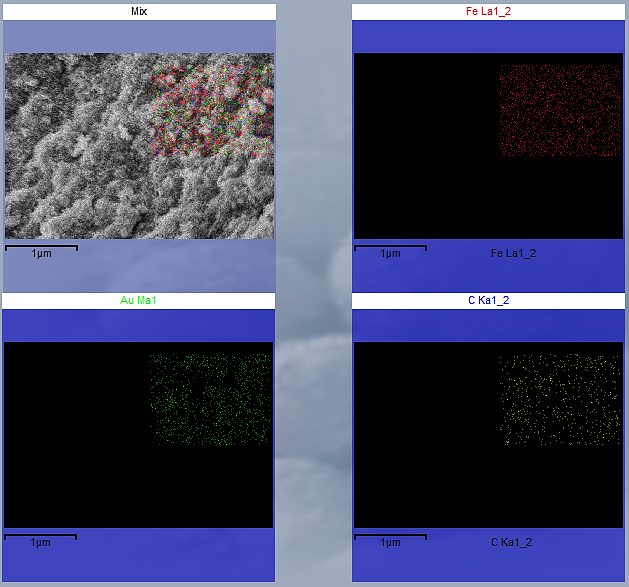


**Fig. S5.** SEM EDS mapping of the composite electrode material (Fe_3_O_4_-Au-IL). Fe, Au, and C are representing Fe_3_O_4_NPs, AuNPs, and ionic liquid, respectively. The color distribution of the elements elucidates their homogeneous distribution on the electrode surface.
